# Redefining the H-NS protein family: a diversity of specialized core and accessory forms exhibit hierarchical transcriptional network integration

**DOI:** 10.1093/nar/gkaa709

**Published:** 2020-09-07

**Authors:** Stephen Fitzgerald, Stefani C Kary, Ebtihal Y Alshabib, Keith D MacKenzie, Daniel M Stoebel, Tzu-Chiao Chao, Andrew D S Cameron

**Affiliations:** Department of Biology, University of Regina, Regina, Saskatchewan S4S 0A2, Canada; Division of Immunity and Infection, The Roslin Institute and R(D)SVS, University of Edinburgh, Edinburgh EH25 9RG, UK; Department of Biology, University of Regina, Regina, Saskatchewan S4S 0A2, Canada; Department of Biology, University of Regina, Regina, Saskatchewan S4S 0A2, Canada; Institute for Microbial Systems and Society, University of Regina, Regina, Saskatchewan S4S 0A2, Canada; Department of Biology, University of Regina, Regina, Saskatchewan S4S 0A2, Canada; Institute for Microbial Systems and Society, University of Regina, Regina, Saskatchewan S4S 0A2, Canada; Department of Biology, Harvey Mudd College, Claremont, CA 91711, USA; Department of Biology, University of Regina, Regina, Saskatchewan S4S 0A2, Canada; Institute of Environmental Change and Society, University of Regina, Regina, Saskatchewan S4S 0A2, Canada; Department of Biology, University of Regina, Regina, Saskatchewan S4S 0A2, Canada; Institute for Microbial Systems and Society, University of Regina, Regina, Saskatchewan S4S 0A2, Canada

## Abstract

H-NS is a nucleoid structuring protein and global repressor of virulence and horizontally-acquired genes in bacteria. H-NS can interact with itself or with homologous proteins, but protein family diversity and regulatory network overlap remain poorly defined. Here, we present a comprehensive phylogenetic analysis that revealed deep-branching clades, dispelling the presumption that H-NS is the progenitor of varied molecular backups. Each clade is composed exclusively of either chromosome-encoded or plasmid-encoded proteins. On chromosomes, *stpA* and newly discovered *hlpP* are core genes in specific genera, whereas *hfp* and newly discovered *hlpC* are sporadically distributed. Six clades of H-NS plasmid proteins (Hpp) exhibit ancient and dedicated associations with plasmids, including three clades with fidelity for plasmid incompatibility groups H, F or X. A proliferation of H-NS homologs in Erwiniaceae includes the first observation of potentially co-dependent H-NS forms. Conversely, the observed diversification of oligomerization domains may facilitate stable co-existence of divergent homologs in a genome. Transcriptomic and proteomic analysis in *Salmonella* revealed regulatory crosstalk and hierarchical control of H-NS homologs. We also discovered that H-NS is both a repressor and activator of *Salmonella* Pathogenicity Island 1 gene expression, and both regulatory modes are restored by Sfh (HppH) in the absence of H-NS.

## INTRODUCTION

Bacterial nucleoid associated proteins (NAPs) are abundant DNA-binding proteins that perform the dual functions of regulating gene expression and shaping higher-order chromosome structures. The heat-stable nucleoid-structuring protein (H-NS), also referred to as the histone-like nucleoid-structuring protein, is an archetypal NAP best studied as a repressor of gene expression in *Escherichia coli*, *Salmonella enterica* and other Gram-negative bacteria (1). H-NS performs two core cellular functions: it represses the expression of hundreds of gene targets, and it forms scaffolds that constrain chromosomal microdomains (2–5). This dual functionality allows H-NS to repress gene expression through a classical mechanism of competing with activator proteins for DNA binding sites in gene promoters, or H-NS can repress transcription at a broader level by restructuring regions of DNA to sequester gene promoters (6–9).

H-NS regulates a wide range of phenotypes in *E. coli* and *Salmonella* in response to environmental parameters, including temperature, osmotic pressure, metal ion concentrations and pH (10–13). H-NS exerts global transcriptional control by polymerizing along extended stretches of DNA and forming higher-order structures that connect disparate DNA loci through protein bridges. H-NS responds to physico-chemical factors by undergoing conformational changes in filaments and bridges, thus transducing gene regulatory signals by modifying DNA topology and promoter activity (7,14–16). In addition to operating as a regulatory nexus of transcriptional activity and cellular physiology, H-NS maintains cellular fitness by preventing intragenic transcription from promoter-like sequences (17) and by silencing the expression of horizontally-acquired AT-rich genes (2,5,14,18–21). The ‘xenogeneic silencing’ of horizontally acquired genes has been observed in *S*. *enterica* serovar Typhimurium, where H-NS represses the horizontally-acquired *Salmonella*Pathogenicity Islands (SPI)-1 and SPI-2 that mediate invasion of host tissues during infection (2,6,22).

H-NS belongs to a large family of functionally and structurally similar proteins found in a diversity of Gram-negative bacteria, including all families of γ-proteobacteria, and in some genera of α- and β-proteobacteria, including *Rhodobacter* (α), *Bordetella* (β) and *Burkholderia* (β) (23,24). In genomes that encode two or more H-NS homologs, amino acid sequence and structural conservation allow homologs to form protein–protein contacts (25,26). Conversely, conservation of DNA binding properties causes homologs to compete for overlapping DNA binding sites (3,27,28). The resultant diversity of hetero-oligomers and DNA binding modes creates interconnected gene regulatory networks that are modulated by the relative concentrations of H-NS and its homologs (29).

The best characterized H-NS homolog, StpA (Suppressor of mutant td phenotype), shares 58% amino acid identity with H-NS and a common two-domain structure (30). StpA binds at and regulates many of the same genes as H-NS, it can form homodimers, heterodimers and oligomers with H-NS through N-terminal interactions, and StpA can form protein filaments and bridges like H-NS (27,28,31–34). Although initially considered a molecular back-up for H-NS, the StpA regulons in *E. coli* and *Salmonella* have since been shown to contain genes outside the H-NS regulons (27,28). StpA has additional RNA chaperone activity, is more thermostable, and binds DNA with greater affinity than H-NS (31,35,36). Distinct and overlapping biological properties with H-NS have also been described for another chromosomal homolog, Hfp (H-NS family protein). Hfp was identified in uropathogenic *E. coli* (UPEC), where it is required for normal growth and repression of the *bgl* and K5 capsular determinant genes, and is a negative regulator of *hns* expression (26). Little is known about Hfp function as it is rare and sporadically distributed in *Escherichia* species.

Sfh (*Shigella flexneri*H-NS-like protein) is an H-NS homolog encoded by pSf-R27, a large, self-transmissible, IncHI1 plasmid (37,38). Three-way protein-protein interactions between H-NS, StpA and Sfh have been observed in *Shigella flexneri*, and Sfh was shown to restore motility and maintain repression of *proU* and *bgl* in an *hns* mutant background (25). Sfh was found to bind at and regulate a defined sub-set of H-NS regulated genes with lower AT content than those preferred by H-NS (3). In *S*. Typhimurium, Sfh can act as a ‘stealth factor’ that protects host cells from uncontrolled expression of chromosomal and plasmid-encoded genes caused by H-NS binding to newly arrived plasmid genes (39). Thus, stealth function is explained as an extension of the xenogeneic silencing exhibited by H-NS (20).

A robust phylogenetic analysis is required to resolve the diversity, origins, and functional relationships of H-NS family proteins. Despite an increasing number of mechanistic studies of H-NS homologs in *Escherichia*, *Salmonella* and other genera of Enterobacteriaceae, the phylogenetic distribution of these homologs is largely unclear. Also problematic is that a mix of manual and automated genome annotations according to top BLAST hits has resulted in H-NS homologs with multiple names in use (e.g. Hfp and HNS2) (26,40), or multiple homologs in a genome all being classified as H-NS. Thus, gene and protein names may not accurately reflect evolutionary and functional relatedness. Furthermore, the lack of rigorous phylogenetic classification of H-NS homologs raises the possibility that distinct clades of H-NS homologs have gone undetected.

Enterobacterales presents an ideal bacterial order in which to study the evolution and interaction of H-NS family proteins as all members appear to encode a chromosomal H-NS protein, with a diversity of homologs encoded chromosomally and on large self-transmissible plasmids (23,24,41). Here, we present a delineation of the H-NS protein family using model genomes from four bacterial families, Enterobacteriaceae, Erwiniaceae, Pectobacteriaceae and Yersiniaceae. Three chromosomally-encoded clades correspond to the established family members H-NS, StpA and Hfp. The phylogeny adds two newly identified clades of chromosomal proteins that we have collectively dubbed Hlp (H-NS-like protein) with a modifier to reflect localization on chromosomes (HlpC) or allegiance to the predominant host genus *Pantoea* (HlpP). An additional six clades are strictly constrained to plasmids, which we named H-NS plasmid proteins (Hpp) and classified according to incompatibility groups F (HppF), H (HppH) and X (HppX), or according to taxonomic allegiance in the three clades where Inc groups could not be predicted, *Pantoea* (HppP), *Erwinia* (HppE) and *Rahnella* (HppR). To examine how homologs modulate H-NS function, we tested how the presence or absence of discrete H-NS subtypes impact the proteome and gene expression in a strain of *Salmonella* Enteritidis that contains up to four H-NS homologs. This revealed hierarchical control of gene expression with H-NS at the apex as a strong repressor of virulence gene expression and a repressor of *hfp* and *stpA* expression.

## MATERIALS AND METHODS

### Genome sequence analysis

The bioinformatic workflow used to identify H-NS homologs in Enterobacterales genomes is outlined in Supplementary Figure S1. At the project start, plasmid-encoded H-NS proteins were initially identified in 2014 by BLASTP searching the NCBI Microbial Plasmid database using *E. coli* K-12 strain W3110 H-NS (BAA36117.1) and StpA (BAA16535.1) and the IncHI1 plasmid-encoded Sfh (AAN38840.1) as query sequences (Supplementary Table S2). For each strain identified to encode a plasmid-borne H-NS homolog, the whole genome sequence was searched for all additional homologs. A separate strategy was used to find homologs of Hfp (ABG69928.1) from *E. coli* UPEC strain 536, which was identified on chromosomes of several non-*E. coli* species from our initial searches (Supplementary Figure S1). All Enterobacterales genomes were searched using the protein sequence as a BLASTP query to identify any Hfp homologs. Any genomes found to carry Hfp homologs were subsequently searched using H-NS, StpA, and Sfh as query sequences to find any other H-NS homologs that occur in Hfp-containing genomes, as described above.

In all BLAST searches, truncated H-NS homologs such as Ler or Hha (7) that consist solely of an N- or C-terminal domain were excluded from consideration by restricting searches to proteins with >50% amino acid identity to H-NS and a BLAST cut-off value of *E* < 10^−10^.

The Plasmid Database (PLSDB) (https://ccb-microbe.cs.uni-saarland.de/plsdb/) (42) version 2019_10_07, containing 18 457 complete plasmid sequences, was queried using *Salmonella enterica* Typhimurium SL1344 H-NS (CBW17777.1). The resulting list of 465 homologs was curated as follows: removal of non-Enterobacterales species (39 homologs), removal of rare Enterobacterales genera not included in this study (*Cedecia* (one homolog), *Leclercia* (seven homologs), *Phytobacter* (one homolog), *Plesiomonas* (two homologs) and *Raoultella* (four homologs)), and removal of two plasmids determined to be mis-assembled contigs that contain large segments of chromosomal genes including *hns* and *stpA* (Supplementary Table S3). The remaining 409 plasmid-encoded H-NS homologs (Supplementary Table S3) contained hundreds of identical sequences, which were collapsed to yield 54 unique H-NS homolog sequences for inclusion in the phylogenetic analysis.

### Phylogenetic analysis

Multiple sequence alignment of all H-NS and H-NS homolog sequences was performed using MAFFT with default settings as part of the Max-Planck institute Bioinformatics toolkit (43) after identical protein sequences were collapsed into single representative sequences. Protein alignments were imported into MEGA6 (44) and maximum-likelihood (ML) trees were constructed using the LG+G amino acid substitution model and 500 bootstrap replicates. Tree analysis and visualisation was carried out using iTOL (45) and FigTree v1.4.2 (A. Rambaut, FigTree [http://beast.bio.ed.ac.uk/FigTree]). The reference genome phylogeny for Enterobacterales was provided by the Pathosystems Resource Integration Center (PATRIC) (www.patricbrc.org) (46).

### Sequence motif analysis

The multiple alignment of all H-NS homologs was subdivided into the subtypes identified as clades in the phylogenetic analysis. WebLogo3 (http://weblogo.threeplusone.com) (47) was used to count amino acids at each position, and these counts were subsequently divided by the amino acid composition in the H-NS consensus to determine percent conservation and plotting as heatmaps. Sequence logos were generated by WebLogo3 with the units field set to ‘probability’.

### Bacterial strains and plasmids

Bacterial strains used for experiments in this study, their genotypes and source, where applicable, are listed in Supplementary Table S1; all are derivatives of *S*. Enteritidis strain EN1660 (48). Unless otherwise stated bacterial strains were cultured with aeration (200 rpm) in Luria broth (LB), also referred to as Lysogeny broth (LB), (10 g l^−1^ tryptone, 5 g l^−1^ yeast extract, 10 g l^−1^ NaCl; pH 7.0) at 37°C. Kanamycin, gentamicin, ampicillin and chloramphenicol were used at concentrations of 50, 50, 50 and 20 μg ml^−1^ respectively.

### Strain construction and DNA manipulation

To delete the *stpA* and *hfp* open reading frames in *S*. Enteritidis EN1660, the *cat* and *neoR* resistance genes were PCR amplified from plasmids pKD3 and pKD4 followed by λ-red mediated recombination to create Δ*stpA* and Δ*hfp* deletion mutants (49). Mutations were transduced to a fresh EN1660 background by generalized P22 transduction and confirmed by DNA sequencing. The Δ*hns* mutation was generated previously in *S*. Typhimurium LT2a (50) and was introduced to *S*. Enteritidis EN1660 by P22 transduction. The *hns* gene sequence and promoter region are identical between LT2a and EN1660; further, transduction from *S*. Typhimurium to *S*. Enteritidis is not expected to result in significant genetic changes because the 10 kbp flanking *hns* has 99.3% identity between the two strains, with only 72 single nucleotide variants across the region. Plasmid pSfR27 was conjugated into *S*. Enteritidis EN1660 and derivative as described previously (39). Briefly, donor (*S*. Typhimurium SL1344 pSfR27) and recipient strains were grown separately overnight at permissive temperature (25°C) without shaking. Donor and recipient strains were mixed 1:5 and 20 volumes of LB broth added. Mixed cultures were incubated statically at 25°C for 24 h to allow conjugation. *S*. Typhimurium SL1344 has a mutation in *hisG* so cannot grow on M9 minimal medium without histidine supplementation, allowing selection of pSfR27 positive *S*. Enteritidis EN1660 colonies on M9 minimal medium with gentamicin.

### Genomic DNA extraction and purification, genome sequencing, and sequence alignments

*S*. Enteritidis EN1660 Δ*hns* was inoculated from frozen glycerol stock onto Lennox agar (10 g l^−1^ tryptone, 5 g l^−1^ yeast extract, 10 g l^−1^ NaCl, 1.5% agar; pH 7.0) and grown overnight at 37°C. Colonies were used to inoculate 5 ml Lennox broth and the culture was incubated for 22 h at 37°C with agitation at 200 RPM. 200 μl of the broth culture (optical density of 1.30 at 600 nm) was collected and used as input for genomic DNA extraction. The extraction was performed using the Rapid Bacterial Genomic DNA Isolation Kit (Bio Basic; BS8225), and DNA eluted with Tris-EDTA solution (10 mM Tris–HCl, 1 mM EDTA; pH 8.0). A genomic DNA library was prepared for sequencing using the NEB Ultra II FS DNA Library Prep Kit for Illumina (New England BioLabs; E7805S), following the procedure for samples with ≤100 ng of input DNA. As part of library preparation, five PCR cycles were used to enrich for adaptor-ligated DNA fragments and the sample was indexed using the NEBNext Multiplex Oligos for Illumina, Index Primers Set 1 (New England BioLabs; E7735S). The library was sequenced using the Illumina MiSeq platform and a Reagent Nano Kit V2 (paired end, 500 cycles).

Demultiplexed sequences were filtered for PhiX sequences using bbduk (51) and the *Enterobacteria* phage phiX174 *sensu lato* reference sequence (RefSeq: NC_001422.1). Filtered sequencing reads were then quality trimmed using Trimmomatic version 0.39 (52) and the following parameters: ILLUMINACLIP:TruSeq3-SE.fa:2:30:10:8:TRUE, CROP 250, HEADCROP 10, LEADING 3, TRAILING 3, SLIDINGWINDOW 4:30, MINLEN 36. Trimmed sequences were imported into Geneious Prime 2019. The whole genome sequence of the wildtype strain was previously published and is available at GenBank (LUUA00000000.1) (48).

### Quantitative reverse transcription PCR

RNA was isolated from bacterial cultures during exponential growth (OD_600 nm_ = 0.12–0.15) and stationary phase (OD_600 nm_ = 2.0). 0.2OD_600 nm_ units of bacteria were harvested, held on ice and 2/5 volume of stop solution (95% ethanol/5% phenol) was added. Total RNA was extracted using an EZ-10 Spin Column Total RNA Miniprep kit (Bio-Basic, Canada) and RNA concentration was quantified using a Nanodrop ND-1000. 2 μg of RNA from each sample was DNase treated using Turbo^™^ DNase (Invitrogen^™^) in a 50 μl reaction and 5 μl of DNase treated RNA was then reverse transcribed using a Verso cDNA synthesis kit (Thermo Scientific^™^) to generate cDNA pools. The relative abundance of target mRNA molecules was determined by quantitative reverse transcription PCR (qPCR) using gene specific primer pairs (Supplementary Table S6) and iTaq™ Universal SYBR^®^ Green Supermix (Bio-Rad). Quantification of mRNA was achieved using an internal calibration curve generated from serially diluted genomic DNA of known quantity.

### Mass spectrometry

Cells from exponentially growing wildtype and mutant *S*. Enteritidis EN1660 cultures (100 ml) were harvested at OD_600 nm_ 0.12–0.15, then lysed with bead beating. Proteins were extracted using acetone precipitation overnight at −20°C. After resolubilization in 50 mM ammonium bicarbonate buffer, 50 μg of protein sample was digested with trypsin (Sigma-Aldrich). After drying, the resulting peptides were spiked with 40 fmol/μl rabbit phosphorylase B in running buffer (0.1% formic acid, 3% acetonitrile) before analysis with a Synapt G2 HDMS (Waters) coupled to a Nanoacquity (Waters) nano-LC with an Acquity UPLC T3HSS column (75 mm × 200 mm). The separation was conducted with a gradient from 3% acetonitrile/0.1% formic acid to 45% acetonitrile/0.1% formic acid at a flow rate of 0.3 μl/min. A total of 1 μg digest was injected in each run and eluting peptides were analysed in positive data-independent-acquisition mode (MSE) with 1s scan time. In low-energy MS mode, data were collected at a collision energy of 4eV. High-energy collision energy was ramped between 18 and 42 V. Leucine enkephaline was measured as lock mass every 30s to maintain mass accuracy throughout the run. The resulting spectra were analysed with the ProteinLynx Global Server (PLGS) v.3.02 and searched against the UniProt *S. enterica* reference proteome (UP000001014, accessed January 2016). Protein abundance was measured together with the identification using phosphorylase B as calibrant. The false discovery rate of protein identifications was set to 4% per run. i.e. proteins identified in all three biological replicates had an expected false discovery rate of <0.001%.

## RESULTS

### H-NS homologs are differentiated into deep-branching clades

To establish a robust phylogeny of H-NS homologs that co-occur in Enterobacterales, we considered only bacterial strains encoding plasmid-borne H-NS homologs identified in the NCBI Microbial Plasmid database, and belonging to the families: Enterobacteriaceae, Erwiniaceae, Pectobacteriaceae and Yersiniaceae. These selection criteria yielded 29 plasmid-encoded H-NS homologs in 15 genera (Supplementary Table S1). For each of these strains, BLAST was used to identify all other H-NS homologs in the genome. In addition to the well-characterized homologs StpA and Sfh, this workflow identified proteins identical or highly similar (>90% amino acid identity) to the recently discovered protein Hfp. Hfp was previously only identified in uropathogenic *E. coli* (UPEC) species (26), prompting us to perform a second search of complete Enterobacterales genomes for proteins with >90% amino acid identity to Hfp. In cases where identical protein sequences were encoded by two or more genomes of the same species, a single representative genome was retained for analysis. This yielded 38 additional Hfp sequences in 30 strains. Other genera, *Hafnia, Edwardsiella, Morganella, Photorhabdus, Xenorhabdus* and *Providencia*, did not encode Hfp, StpA, or any plasmid-borne H-NS proteins, so were not further considered in this study. Altogether, the dataset contained 193 protein sequences from 15 genera of Enterobacterales (Tables S1 and S2), of which 116 were unique sequences (88 chromosomal and 28 plasmid).

**Figure 1.**
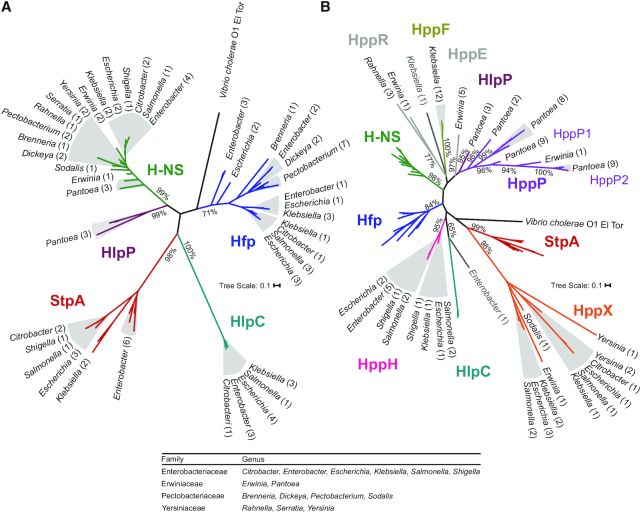
Phylogenetic classification of H-NS family proteins in Enterobacterales. (**A**) Chromosomal-encoded H-NS homologs. (**B**) Chromosomal and plasmid-encoded H-NS homologs. The protein clades are categorized as: H-NS (green), StpA (red), Hfp (blue), HlpC (teal), HlpP (purple), HppP (magenta), HppX (orange), HppH (pink), HppE (grey), HppR (grey) and HppF (gold). The bacterial genera that encode the homologs are indicated at the branch tips, and the number of representative protein sequences are in brackets. Clusters of branches that are too small to resolve in the figure are coloured with grey background to improve connections between branches and names. Rooted phylogenies with each branch and species name are provided in Supplementary Figure S2. Phylogenies are derived from maximum likelihood inferences of evolutionary relationships, and the scale bar indicates the number of substitutions per site. Bootstrap scores are shown for ancestral branches that define the clades and sub-clades described in the text.

Phylogenetic relationships between H-HS homologs were inferred using maximum likelihood. Initial consideration of chromosomally-encoded proteins revealed five evolutionarily distinct protein clades, each with bootstrap support over 70% (Figure 1A and Supplementary Figure S2A). The phylogeny in Figure 1A is unrooted to facilitate visualization of each high-confidence homolog type, or ‘clan’ (53), using a coloured branch, while simultaneously deemphasizing the tree trunk where branching order cannot be determined due to low bootstrap support. Each clan in the unrooted phylogeny forms a clade in a tree rooted with *Vibrio cholerae* H-NS (Supplementary Figure S2A). Three clades correspond to each of the previously described chromosomal H-NS homologs: H-NS (green), StpA (red) and Hfp (blue). All genomes in this analysis encode H-NS, whereas StpA and Hfp have restricted distributions that are examined in detail below. A newly resolved clade was detected in the Enterobacteriaceae genera *Escherichia*, *Enterobacter*, *Klebsiella* and *Salmonella*, which we named ‘HlpC’ for H-NS-like protein on chromosomes. Another newly resolved clade is comprised of proteins present only in the genus *Pantoea*, which we named ‘HlpP’ for H-NS-like protein in *Pantoea*.

### Plasmid-borne H-NS homologs have diverse origins

Plasmid-borne H-NS homologs have been identified on a diversity of plasmids, but the evolutionary origins and biological functions of these homologs remain largely unexplored. To test the simplest hypothesis that plasmid-borne H-NS homologs arose by plasmid capture of chromosomal *hns*, a second phylogenetic analysis was conducted that included: all chromosomal H-NS homologs described above, the 29 plasmids sequences from the NCBI Microbial Plasmid database, and 54 additional H-NS homolog sequences identified in the Plasmid Database (PLSDB). Figure 1B shows the expanded phylogeny of H-NS homologs with plasmid-encoded proteins included. Importantly, the addition of a large number of new sequences did not affect the cohesion of the chromosomal clades in Figure 1A. Moreover, none of the plasmid forms occur in the H-NS clade, indicating that none of the plasmid forms are descended from chromosomal H-NS.

Six new clades are composed exclusively of plasmid-borne sequences, and we have dubbed proteins in each of these clades as H-NS plasmid proteins (Hpp) (Supplementary Table S4). Among these, only the protein Sfh has been characterized (25,37). Sfh has been identified in *Shigella* and *Salmonella* on the plasmids pSf-R27 and pR27, respectively, that belong to plasmid incompatibility (Inc) group H (25,54,55). The other proteins in this clade are also encoded by IncH plasmids, thus we refer to members of this clade as HppH. Two additional protein clades correspond to Inc groups X and F, for which we have named the clades HppX and HppF, respectively (Figure 1B). HppX contains the greatest sequence diversity of any H-NS homolog clade; besides H-NS, it is the only homolog found in all four bacterial families being considered here. In contrast, HppF is found only on plasmids in *Klebsiella* in this analysis. The HppF, HppH, and HppX clades each branch from the trunk of the unrooted phylogenetic tree (black in Figure 1B); this distinction from the chromosomal homolog clades suggests these plasmid forms are unlikely to have originated from duplication and divergence of a chromosomal gene in the Enterobacterales.

Three additional clades of Hpp are present in the phylogeny, but these could not be classified according to plasmid replicons due to the absence of recognizable lnc elements. Instead, we named these clades according to the predominant bacterial hosts: *Erwinia* (HppE), *Pantoea* (HppP) and *Rahnella* (HppR) (Figure 1B). A single H-NS homolog sequence encoded on two plasmids in PLSDB was connected by a deep branch (65% bootstrap support) to HlpC, which was too distant to include in the HlpC clade (Figure 1B); this branch is represented by pASM1 in Supplementary Figure S2B. A single H-NS homolog on plasmid pKOX-ea2b branches closest to HppF, but the long branch and very low bootstrap support preclude its inclusion in the HppF clade (Figure 1B).

### Evolutionary origins of chromosomal H-NS homologs

H-NS is present in all genomes analysed (Supplementary Table S1). Low bootstrap support of Pantoea and Erwinia H-NS branches indicates that branching order within the H-NS clade is not well resolved (Supplementary Figure S2A). Nevertheless, when accounting for the low confidence branching order, the phylogeny of core H-NS is congruent with the phylogeny of Enterobacterales genomes (Supplementary Figure S3A). The simplest interpretation of the phylogenies is that H-NS was present in the last common ancestor of all Enterobacterales and was never lost nor replaced over evolutionary time, consistent with its central role in cellular homeostasis. Further, there is no evidence of horizontal transfer of H-NS between genera.

The chromosomally-encoded homolog StpA is present only in the Enterobacteriaceae, indicating a singular origin or acquisition event in the common ancestor of *Escherichia*, *Salmonella*, *Klebsiella*, *Enterobacter* and *Citrobacter*. To improve the resolution of the *stpA* evolutionary history in the Enterobacteriaceae, maximum likelihood phylogenies for StpA and H-NS were compared to whole-genome phylogenies (Supplementary Figure S3B). Strains from each genus containing StpA, *Escherichia*, *Salmonella*, *Klebsiella*, *Enterobacter* and *Citrobacter*, formed strongly supported monophyletic clusters (Bootstrap value > 95%). Although the StpA cladogram has lower resolution than the genome phylogeny, the StpA, H-NS and whole genome phylogenies are congruent (Supplementary Figure S3B). It was previously inferred that *stpA* originated from *hns* duplication and divergence in a recent ancestor of *Salmonella* and *E. coli* (24,56). However, if *stpA* arose by duplication of *hns* within the Enterobacteriaceae, the StpA clade would be found as a distinct branch within the H-NS clade, which it is not (Figure 1A). Instead, phylogenetic evidence suggests that *stpA* was acquired by the common ancestor of *Escherichia*, *Salmonella*, *Citrobacter*, *Enterobacter* and *Klebsiella* through horizontal gene transfer from an unidentified donor outside of Enterobacteriaceae. Conservation of the protein in all extant members of these genera suggests it acquired (or already bestowed) a core genetic function at its inception, preventing its loss from Enterobacteriaceae as the bacterial family diversified into a very wide range of habitats over many tens of millions of years.

The evolutionary history of Hfp is more obscure. Of the 15 genera included in our study, eight contain Hfp (Supplementary Table S1). Within these genera only some strains contain Hfp, consistent with the original discovery of *hfp* in a small number of *E. coli* strains (26,57). A notable feature of Hfp is that it is the only protein we found encoded multiple times on some chromosomes, as observed in some strains of *Escherichia*, *Dickeya* and *Pectobacterium* (Supplementary Table S1). The incongruence of the Hfp phylogeny compared to those of H-NS and StpA is most simply explained by horizontal transfer between genera and even between families Pectobacteriaceae and Enterobacteriaceae (Supplementary Figure S3B). In *E. coli*, *hfp* was consistently found within a genomic island inserted upstream of *asnW* tRNA, as noted in uropathogenic *E. coli* (26,57), or *serU* tRNA. In *Salmonella*, *hfp* was also consistently located within a genomic island. A recent study investigating the region of difference 21 (ROD21) of *Salmonella* Enteritidis led to the classification of a group of Enterobacteriaceae-associated ROD21-like (EARL) genomic islands (58); 33 of 54 EARL islands encode H-NS homologs, and we confirmed by sequence alignment that all cases are Hfp.

### The genetic contexts of H-NS plasmid proteins

**Figure 2.**
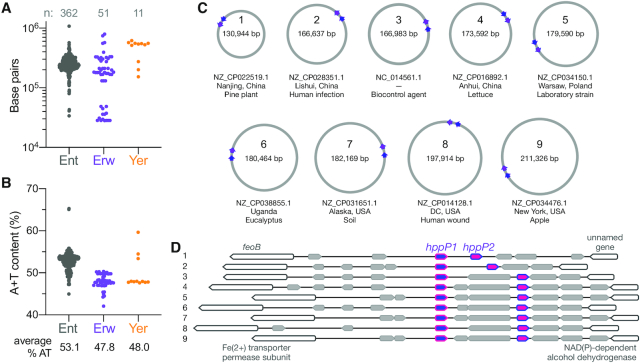
Plasmids containing H-NS homologs. (**A**) Size distribution of plasmids sequences in PLSDB that encode H-NS homologs. Bacterial families are indicated as Ent, Enterobacteriaceae; Erw, Erwiniaceae and Yer, Yersiniaceae. (**B**) Average AT content of plasmids sequences in PLSDB that encode H-NS homologs. (**C**) *Pantoea* plasmids encoding HppP1 and HppP2. In the absence of annotated origins of replication, plasmids are oriented according to nucleotide numbering in GenBank records. (**D**) Gene content flanking *hppP1* and *hppP2*. Genes *feoB* and alcohol dehydrogenase (white) are conserved on all nine plasmids and were used as boundaries for comparing the genetic loci.

A rapidly growing number of complete plasmid sequences enables expanded surveys for H-NS homologs and their genetic context. It has been observed that plasmids containing homologs of H-NS and other nucleoid associated proteins are large (usually over 100 kb) and AT-rich (average 54% AT) (59). In late-2019, the PLSDB contained 464 plasmids sequences with an H-NS homolog, of which 424 occur in bacteria in the order Enterobacterales. The majority, 362, are from Enterobacteriaceae, with a median size of 245 kb and median AT of 53% (Figure 2A and B). The much smaller number of representatives from the Erwiniaceae and Yersiniaceae were also large (median 180 and 542 kb, respectively), but much lower average AT (median 48% in both families).

Eleven plasmids in PLSDB encode two copies of HppP and one plasmid encodes two copies of HppE (Supplementary Table S5), making this the first observation of two H-NS homologs encoded on the same plasmid. In one case, the identical HppP occurs twice at distant locations in different genetic contexts on a large plasmid (NZ_CP014208.1) in *Pantoea ananatis*. In the other case of a duplicate protein, HppE is part of a ∼10 kb region that is duplicated on the ∼48 kb plasmid pEP48 (NZ_CP023568.1) in *Erwinia pyrifoliae*. Figure 2C examines the nine plasmids that encode two distinct HppP sequences. The host *Pantoea* species were isolated from plants, human infections, or laboratory stocks in diverse locales around the world (Figure 2C). The two *hppP* genes are consistently located close to one another on plasmids that range in size from 131 to 211 kb. Each of the paired *hppP* genes represents distinct sub-clades, HppP1 and HppP2 in Figure 1B, that have 96% and 100% bootstrap support, respectively. This phylogenetic differentiation and the diversity of plasmids and bacterial hosts harbouring these paired HppP suggests a long evolutionary history of linkage. The resilience of the HppP1 and HppP2 pairing is confirmed by examining their genetic loci. In all nine plasmids, the two genes are linked to *feoB* and an unnamed gene encoding an alcohol dehydrogenase. Yet, the variable number of genes flanking and between *hppP1* and *hppP2* indicates that the pairing has persisted despite a significant amount of genetic change (Figure 2D). A closely related protein is found alone in a plasmid in *Erwinia* (NZ_LN907828.1) (Figure 1B).

### Amino acid divergence at functionally important positions

**Figure 3.**
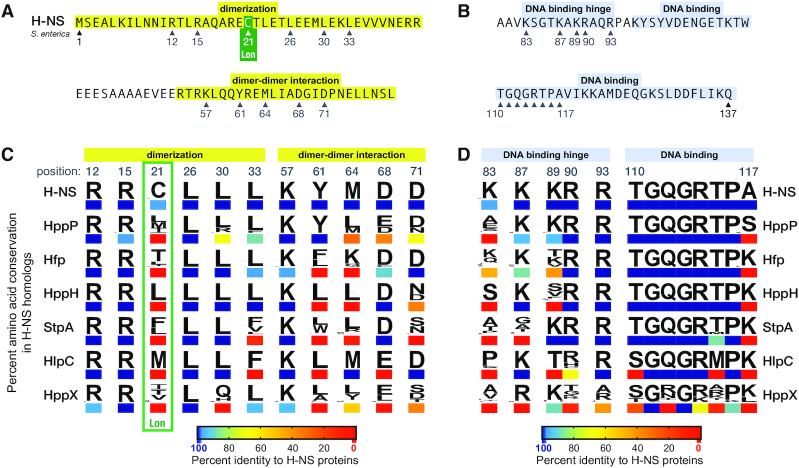
Motif analysis of amino acid conservation and divergence between clades of H-NS homologs. The *S. enterica* H-NS amino acid sequences of the N-terminus (**A**) and C-terminus (**B**) are presented with functional domains highlighted. Amino acid positions where substitutions compromise specific protein functions are numbered in grey; the studies that identified these amino acids positions are cited in Supplementary Table S7 along with additional experimental evidence based on deletion mutations. (**C**, **D**) Frequency logos and heatmap colouring to compare the percent identity of amino acids to the H-NS consensus sequence. The clades with small numbers of protein sequences (HppE, HppF and HppP) were not considered due to the low complexity of sequence data.

We hypothesized that H-NS clades will possess distinct amino acids at positions involved in DNA binding and protein-protein interactions to (i) perform distinct functions and (ii) prevent deleterious interference with H-NS. Hence, we predicted sequence divergence to be concentrated at locations where functional differentiation can arise, while mutations will be resisted at positions required for core functions and inter-clade interactions. H-NS can be functionally subdivided into four functional domains: the N-terminal dimerization and dimer-dimer interaction domains (Figure 3A), and the C-terminal DNA binding hinge and DNA binding domains (Figure 3B). The specific amino acid positions in H-NS for which biological functions have been experimentally determined are indicated by numbered arrows (Figure 3A & B).

Previous studies found that H-NS, StpA, and Sfh have similar DNA biding properties, and both StpA and Sfh can heterodimerize with H-NS (29). However, the biochemical properties and interactions of the new homologs detected in the present study remain unexplored. Amino acid positions with experimentally determined functions are almost 100% conserved in the H-NS clade. The two exceptions are the Lon protease target site Y21 in 5% of H-NS sequences (Figure 3C) and DNA binding hinge K83 in 5% of H-NS sequences (Figure 3C and D). A broader consideration of residue conservation across all H-NS homolog sequences shows that the strong conservation of key residues in H-NS is not shared across the whole protein family (Supplementary Figure S4).

Amino acid substitutions at 10 sites in the N-terminal domain of H-NS significantly disrupt dimer and/or oligomer formation (5,15,31,60–64). Substitutions at five of these locations predominantly disrupt dimer formation, whereas substitutions at the other five mutations disrupt formation of higher-order multimers (Figure 3A). The dimerization residues (R12, R15, L26, L30 and L33) are 100% conserved in all members of H-NS clade (Figure 3C). These same five residues are highly conserved across the other clades, with all HppH proteins being identical to H-NS. Conversely, in StpA (position 33), HlpC (position 33) and HppX (position 30) clades, all proteins are divergent from H-NS. In the dimer-dimer interaction domain, HppP is the most similar to H-NS whereas the proteins in the other clades are highly divergent from H-NS in at least two positions. Therefore, HppP is predicted to form the most stable heteromers with H-NS whereas all other homolog clades are predicted to have a reduced ability to oligomerize with H-NS. Positions where H-NS homologs consistently differ from H-NS, such as 61, 64, 68 and 71, may cause these homologs to preferentially homo-oligomerize and even hetero-oligomerize with similar homologs while exhibiting reduced affinity for protein-protein interactions with H-NS.

Environmental sensing by H-NS involves conformational changes that reorient ionic interactions between the N-terminal and central dimerization domains (65). These interactions are supported by K57 (65), which is one of the most highly conserved residues across the H-NS family of proteins (Figure 3C; Supplementary Figure S4B). Negatively charged patches within the N-terminal domain and positively charged linker region form ionic bonds that assemble H-NS dimers (16). These charge distributions are conserved across H-NS family proteins (Supplementary Figure S4B).

Recent studies identified a DNA binding hinge in H-NS where positively charged residues K83, K87, K89, R90 and R93 are critical for DNA binding (66,67) (Figure 3B). Replacing positively charged amino acids at these positions reduces H-NS-DNA binding specificity and reduces H-NS repression of gene expression (66,67). All H-NS proteins possess positively charged amino acids (K or R) at these five positions in the hinge (Figure 3D); the only substitution within the clade is 5% of H-NS have R83. The H-NS homolog clades are strikingly divergent in this sub-domain, except at R93, where only HppX is divergent from H-NS.

StpA, Hfp and Sfh (HppH) bind to many of the same chromosomal loci as H-NS in *E. coli* and *Salmonella* (3,27,40). The DNA binding domain (T110–A117) is 100% conserved within the H-NS clade and largely conserved across the homolog clades (Figure 3D). Conservation of the key amino acids within the DNA binding domain suggests that each homolog has the capacity to compete with H-NS for DNA binding sites, or can reinforce DNA binding activity when forming heteromers.

The Lon protease degrades StpA whereas H-NS is protected by the cysteine at position C21 (68). Only members of the H-NS clade have a cysteine at this position (Figure 3B). All other family members, including StpA, have a hydrophobic residue (I, L, V, T or F). This finding suggests that proteins in each clade (HppP, Hfp, HppH, StpA, HlpC and HppX) may be susceptible to Lon proteolysis.

### Crosstalk between H-NS and its homologs

Genomes encoding H-NS and one or more H-NS homologs present ideal systems in which to study transcriptional regulatory network integration and crosstalk between regulons. H-NS is a master regulator that represses expression of its homologs *stpA* (26,69) and *hfp* (26), and H-NS regulates 13% of the *Salmonella* Typhimurium genome compared to the 5% regulated by StpA (28,70). Negative autoregulation is a common theme in bacterial transcription networks as a feedback circuit limits mRNA levels and transcription factor concentration (71,72). Negative autoregulation also promotes network stability and reduces cell-to-cell variation of abundant transcription factors like H-NS (73). The observed amino acid similarities and predicted functional properties of H-NS homologs suggests that they may negatively repress each other's promoters as a facet of their autoregulation. Several studies have measured the relative abundance of H-NS and StpA (74,75) but quantification of additional homologs and an assessment of H-NS and homolog pools when genes are deleted from the system was lacking.

**Figure 4.**
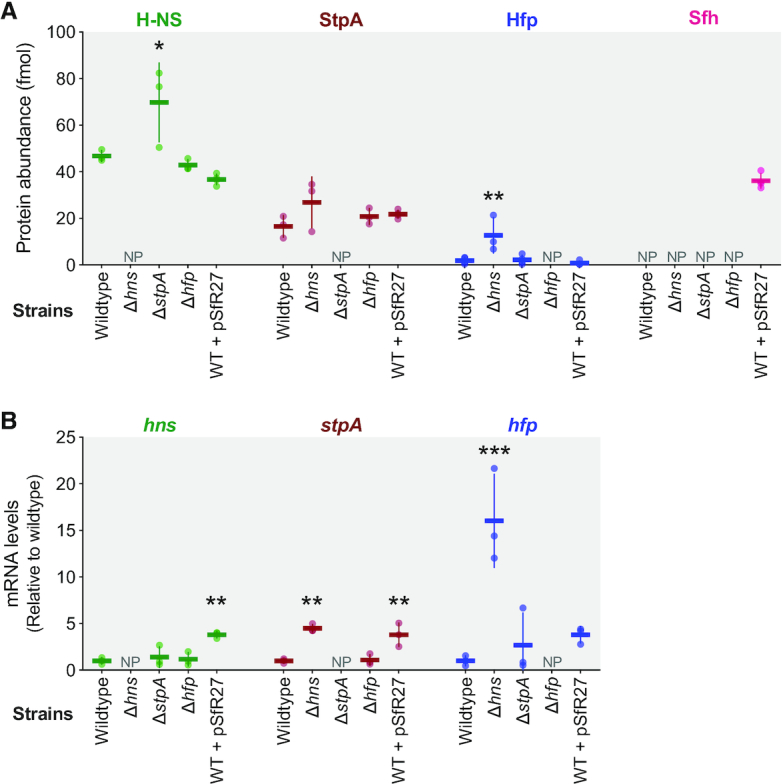
Protein abundance and expression of H-NS family proteins in *S*. Enteritidis EN1660. Cellular protein concentration was quantified by mass spectrometry (**A**) and relative mRNA level was quantified by quantitative PCR (**B**) for H-NS (green), StpA (red) and Hfp (blue) in wildtype strain EN1660 and Δ*hns*, Δ*stpA*, Δ*hfp* mutants during exponential phase. The cellular protein concentration of Sfh was also quantified in wildtype strain EN1660 along with quantification of the impacts of pSf-R27 on *hns*, *stpA* and *hfp* gene expression. The mean and standard deviation of three to four biological replicates is shown. NP: not present (and not detected). Statistical differences were calculated by one-way ANOVA using Dunnett's multiple comparison test of mutants versus wild type. *P* values <0.05 (*), <0.01 (**), <0.001 (***).

We selected *S*. Enteritidis strain EN1660 for proteomic and transcriptomic studies because its chromosome encodes *hns*, *stpA* and *hfp* (48). Mass spectrometry was used to quantify absolute amounts of proteins per 1 μg of injected protein mass, allowing us to compare the relative abundance of each H-NS homolog. In wildtype cells, H-NS (47 fmol) was the most abundant, followed by StpA (17 fmol) and Hfp (2 fmol) (Figure 4A), a ratio of approximately 30:10:1. This ratio of H-NS to StpA is consistent with western blot quantification by previous studies (74–76). We constructed a Δ*hns* mutant and used whole genome sequencing to confirm the precise deletion of *hns* and the absence of compensatory mutations, at least at the single nucleotide polymorphism level. Consistent with the expected de-repression of *stpA* and *hfp* expression in the Δ*hns* mutant, StpA (27 fmol) and Hfp (13 fmol) protein levels increased (Figure 4A). Deleting *stpA* resulted in significantly elevated levels of H-NS, revealing that StpA is a direct or indirect repressor of H-NS. Deletion of *hfp* had no detectable effect on H-NS or StpA protein levels (Figure 4A).

Next we tested whether addition of an exogenous H-NS homolog, Sfh (HppH), would decrease the levels of chromosomally encoded H-NS proteins to maintain a constant total concentration of H-NS family proteins. In nature, Sfh is encoded on the IncHI plasmids R27 in *Salmonella enterica* serovar Typhi and pSf-R27 of *Shigella flexneri* 2a strain 2457T (25,77), where it provides a stealth function by complementing multiple *hns* mutant phenotypes in *E. coli* (37,39). Introducing pSf-R27 to cells resulted in high Sfh levels (36 fmol) approaching concentrations close to H-NS, but this had no detectable effect on the levels of H-NS, StpA or Hfp in wildtype cells (Figure 4A).

Transcript abundance was quantified to identify the transcriptional regulatory connections between H-NS and the multiple H-NS homologs. In exponential phase growth, *hns* expression was not affected by the absence of *stpA* or *hfp*, while addition of *sfh* on pSf-R27 resulted in a 3.8-fold upregulation of *hns* expression (Figure 4B). *stpA* expression increased 4.5-fold and *hfp* expression increased 16-fold in the Δ*hns* mutant compared to wildtype. Deletion of *hfp* had no detectable effect on *hns* or *stpA* transcription. The presence of *sfh* on pSf-R27 caused a 4-fold increase in both *stpA* and *hfp* expression. Altogether, H-NS demonstrated the expected role of repressing *stpA* and *hfp* expression. Increased H-NS protein levels observed in the Δ*stpA* mutant were not reflected in a detectable change in transcription of *hns*, hinting at a post-transcriptional regulatory mechanism that increased translation of *hns* transcripts to supplement the pool of H-NS-like proteins in the absence of StpA.

### Hierarchical control of virulence gene expression

H-NS homologs can be gained and lost over short evolutionary times in the Enterobacterales, prompting us to examine the degrees to which H-NS homologs compete, complement, or compensate for regulatory functions. H-NS is a strong repressor of the genes that encode the type three secretion system and effector proteins in *Salmonella* pathogenicity island 1 (SPI-1) that *Salmonella* uses to initiate invasive infection in a host intestine. HilD is the master activator of SPI-1 gene expression, which is repressed by H-NS binding to the promoter of *hilD* (50,78–80). The *hilD* promoter is also bound by Sfh (3), though whether Sfh represses *hilD* expression has yet to be explored.

**Figure 5.**
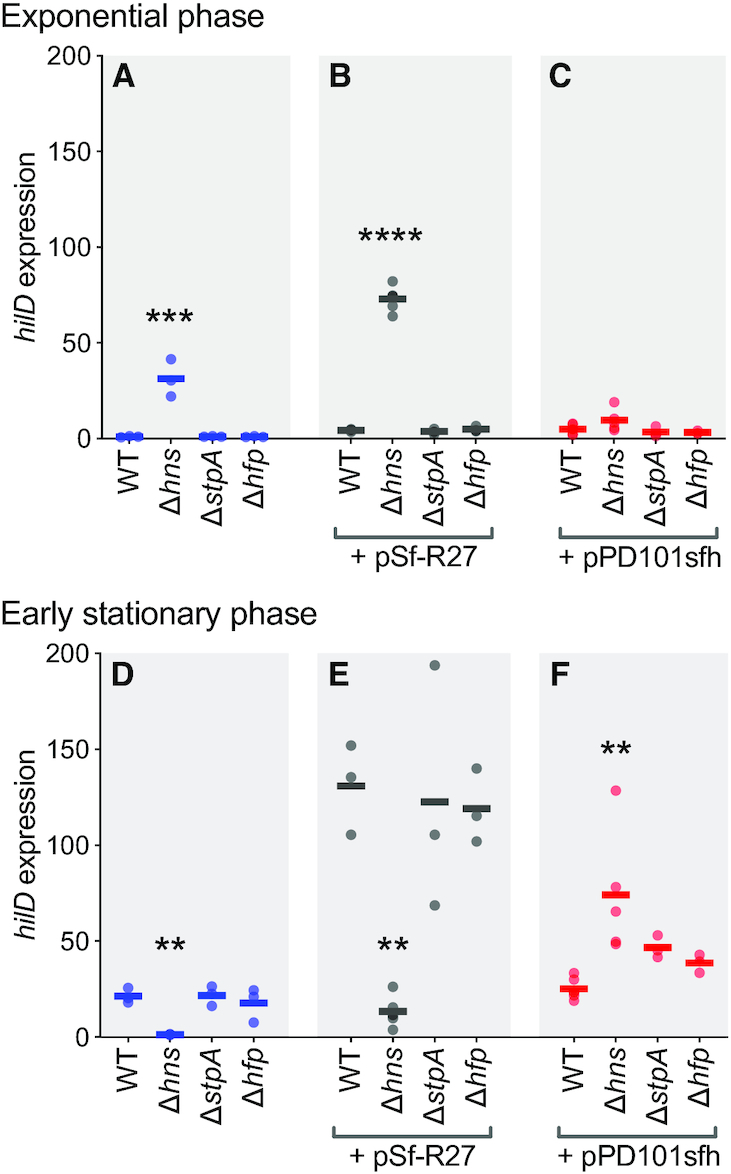
Regulation of *hilD* expression by H-NS family proteins. *hilD* mRNA levels were quantified in wildtype strain EN1660 and Δ*hns*, Δ*stpA*, Δ*hfp* mutants in exponential phase (**A**) and stationary phase (**D**) by quantitative PCR. The influences of plasmid pSf-R27 encoding Sfh (HppH) in its natural context or plasmid pPDsfh encoding Sfh alone on *hilD* expression were also assessed in wildtype EN1660 and each mutant derivative in exponential phase (**B**, **C**) and stationary phase (**E**, **F**). All values are expressed relative to wild type *hilD* expression in exponential phase. Data from three to six biological replicates is shown with the mean represented by a horizontal bar. Statistical differences were calculated by one-way ANOVA of each growth phase and *sfh* background grouping using Dunnett's multiple comparison test of mutants versus wild type. *P* values <0.01 (**), <0.001 (***), <0.0001 (****).

To test for cooperative or competitive regulation of *hilD* expression by combinations of H-NS and H-NS homologs, *hilD* expression was quantified in both exponential growth and stationary phases in wildtype cells and deletion mutants. In wildtype *S*. Enteritidis, *hilD* expression was low in exponential growth, consistent with the well-characterized repression of SPI-1 expression in *S*. Typhimurium (81,82). Deletion of *hns* resulted in a 30-fold increase in expression in this growth phase, whereas deletion of either *stpA* or *hfp* had no detectable effect on *hilD* expression, indicating that H-NS is the dominant repressor of *hilD* in exponential growth (Figure 5A).

Sfh has been previously shown to occupy the *hilD* promoter (3), but production of Sfh by introducing pSf-R27 did not restore repression (Figure 5B). Instead, Sfh in *S*. Enteritidis wildtype and mutant cells caused a modest three- to four-fold increase in *hilD* expression in all genotypes (wildtype, Δ*hns*, Δ*stpA*, Δ*hfp*) in exponential growth phase (Figure 5B); this pattern and degree of transcriptional response is similar to that noted in Figure 4 above when pSf-R27 was introduced to cells for analysis of *hns*, *stpA* and *hfp* expression. Plasmid pSf-R27 also encodes the H-NS N-terminal homolog Hha, which has been shown to interact with H-NS and influence virulence gene expression in *Salmonella* (37,83). To determine if Sfh demonstrates a different regulatory outcome when expressed alone, we introduced a clone of *sfh* under control of its native promoter in plasmid pPD101sfh (39) into *S*. Enteritidis EN1660 wildtype and Δ*hns*, Δ*stpA* and Δ*hfp* mutant strains. Acquisition of Sfh on this alternate plasmid vector resulted in near complete complementation of the loss of H-NS, as observed in the return of *hilD* expression to near wild type (Figure 5C). Thus, in this experimental situation, Sfh is a strong molecular replacement for H-NS, but in its native context, Sfh activity at the *hilD* promoter is somehow tempered by the presence of the many genetic elements on pSf-R27.

The same experiments were conducted in early stationary phase cells, when *hilD* is expected to be naturally induced in laboratory-cultured *Salmonella* (81,82). Consistent with expectations, *hilD* expression in wildtype cells was elevated >20-fold relative to exponential growth (Figure 5D). However, we were particularly surprised to observe that *hilD* was not expressed in Δ*hns* cells in stationary phase (Figure 5D). This highly unexpected result suggests that H-NS functions directly or indirectly as a transcriptional activator of *hilD* in specific environments and genotypes. H-NS is an archetypal repressor of transcription, yet indirect transcriptional activation by H-NS has been observed (84). Addition of pSf-R27 elevated *hilD* expression 6-fold in all genotypes, but did not restore *hilD* expression in the Δ*hns* cells in stationary phase (Figure 5E). Strikingly, addition of Sfh to cells using pPD101sfh significantly increased *hilD* expression relative to wild type, revealing that Sfh can also compensate for the absence of H-NS by up-regulating *hilD* expression (Figure 5F).

The discovery that both H-NS and Sfh contribute to up-regulation of *hilD* expression led us to examine how H-NS and its homologs control the expression of another SPI-1 gene (*sicA*), two SPI-2 genes (*ssrA*, *ssaG*) and a SPI-5 gene that belongs to the SPI-2 regulon (*pipB*). As for *hilD*, H-NS was required for normal repression of all SPI-1 and SPI-2 genes in exponential phase (Supplementary Figure S5A–C). The requirement for *hns* to activate *hilD* expression in early stationary phase was also true for the SPI-1 effector *sicA* (Supplementary Figure S5A). Conversely H-NS acted as a conventional repressor of SPI-2 genes, as *ssrA, ssaG* and *pipB* expression was upregulated in the Δ*hns* mutant. Deletion of *hfp* had no significant effect on expression of SPI-1 or SPI-2 genes, but deletion of *stpA* did result in upregulation of *sicA* and *ssrA* in the presence or absence of pSf-R27. As observed for *hilD*, acquisition of plasmid pSf-R27 caused increased expression of *sicA, ssrA, ssaG*, and *pipB* in all strains in early stationary phase.

## DISCUSSION

Here, we present a phylogenetic reconstruction of the H-NS protein family in Enterobacterales, revealing an unexpected diversity of chromosomal and plasmid-borne homologs. In the scientific literature, H-NS homologs are usually referred to as ‘H-NS paralogs’ due to a presumed evolutionary origin of *hns* duplication and diversification (20,40,85). However, our phylogenetic reconstruction of H-NS family evolution in the Enterobacterales demonstrates that StpA, Sfh and Hfp are homologs but are not paralogs. For example, if *stpA* originated from duplication of *hns* in Enterobacteriaceae, the StpA clade would connect to Enterobacteriaceae H-NS as a branch *within* the green H-NS clade (Figure 1A). The deep branches that distinguish the homolog clades indicate that common ancestry with H-NS very likely predates the origin of the order Enterobacterales.

A parsimonious explanation of the H-NS homolog phylogenies is that each clade was founded by an independent horizontal gene transfer event, with the exception of HppE, HlpP and HppP which may be descended from a single acquisition early in the evolution of the family Erwiniaceae. Similarly, acquisition events can be dated approximately from the distribution of each homolog: the ancestral *stpA* gene entered the common ancestor of the Enterobacteriaceae, after divergence from the other bacterial families examined in this study. Our new understanding of the age and diversity of H-NS homologs underscores a need for further examination of homolog functions and regulons, expanding beyond the pervasive characterization of ‘paralogs’ that are accessory modifiers or mere molecular ‘backups’ to H-NS.

We hypothesize that the H-NS homolog clades demonstrate evolutionary trajectories that reflect the degree to which the homologs integrate into host genomic functions. The apparent conservation of StpA in all extant Enterobacteriaceae and HlpP in all extant *Pantoea* suggests that these two proteins acquired essential functions, ensuring their preservation over evolutionary time. In contrast, the members of most H-NS homolog clades are mobile, resulting in sporadic distributions that can be limited to specific families (HppP, HppE, HppF, HppH, HppR, HlpC) or distributions across multiple bacterial families (HppX, Hfp). Bacterial host ranges may be constrained by the identifiable plasmid replicons (incF, incH, and incX). Similarly, the plasmids without recognizable incompatibility groups may be restricted to specific bacterial species by their replication mechanisms, explaining the phylogenetically constrained distributions of HppP, HppE, HppR and HlpC (Figure 1B).

The diversity and evolutionary histories of plasmid-borne H-NS homologs have not been previously addressed. Our study reveals a rich diversity and broad distribution. The ancient evolutionary association with specific plasmid incompatibility groups may reflect integration with or even control of replicon function. In this model, H-NS homologs may either directly regulate plasmid replication, and/or may contribute structural modification of plasmid DNA topology that is required for the initiation, progression, or resolution of plasmid replication. These hypothetical functions differ from the predominant model of stealth function. First, a stealth function would benefit any foreign DNA silenced by H-NS binding; this would predict H-NS homolog distributions to be more sporadic, not allied to specific plasmid replicon types as observed in our study. Second, the plasmids that encode H-NS homologs in Erwiniaceae and Yersiniaceae display average AT content far lower than in Enterobacteriaceae (Figure 2B), suggesting that AT content is a reflection of plasmid types in these families, not the co-evolution of AT content and H-NS homolog stealth functions.

The chromosomal homologs Hfp and HlpC also demonstrate a propensity for horizontal transfer and loss over short evolutionary periods. HlpC was detected in some members of *Citrobacter*, *Enterobacter*, *Escherichia*, *Klebsiella* and *Salmonella*, but not in genera outside Enterobactericeae (Supplementary Table S1). HlpC has undergone very little change at the sequence level across the five genera, suggesting that all cases represent recent acquisitions and a relatively short evolutionary history in Enterobacterales (Figure 1A). In contrast, the Hfp clade contains extensive diversity, and the phylogeny in Supplementary Figure S3B illustrates how the *hfp* gene has moved horizontally within and between the bacterial families Enterobacteriaceae and Pectobacteriaceae. EARL islands are excisable and mobilizable (20), explaining the horizontal transmission of *hfp* genes. Future research will examine the mobility of *hfp* when it is not associated with EARL islands, as in *Brenneria* and *Dickeya*, in some cases in *Pectobacterium*, and at the serU-tRNA locus in *E. coli*.

The study of Hfp is complicated by the *hfp* gene being independently identified and annotated on three occasions. It was first noted by Williamson and Free in *E. coli* CFT073, who called it *hnsB* (57). *hfp* was identified and studied in *E. coli* 536 (26), and it was only later recognized that *hnsB* and *hfp* were identical, but more recently *hfp* has been named *hns2* in *E. coli* 042 (40). Similarly, automated annotations in the absence of phylogenetic insights are problematic as proteins are usually named according to the most prominent homolog, which has resulted in the annotation of many H-NS homologs simply as ‘H-NS’. For example, all H-NS homologs in the genus *Pantoea* (chromosomal and plasmid) are automatically annotated as ‘H-NS’. Our study underscores how functional and bioinformatic studies in the absence of a phylogenetic understanding result in mis-annotations that hamper scientific communications and restrict scientific advance.

### Implications of H-NS homolog diversity for understanding protein functions

The H-NS N-terminus contains the dimerization and oligomerization domains. When oligomerized, H-NS can form protein filaments along DNA or it can form DNA-protein-DNA bridges that physically connect disparate genetic loci (8,86–88). Both of these binding architectures, filamentation or bridging, impact gene expression and DNA shape by obstructing other protein–DNA interactions and by preventing diffusion of DNA supercoiling (8,88,89). The amino acids that have been experimentally determined to contribute to dimerization are highly conserved across the homolog clades (Figure 3), suggesting that all homologs may be capable of forming heterodimers with H-NS. The dimer-dimer (oligomerization) domain shows greater divergence between clades and within clades, which could reflect diversifying selection that reduces or prevents H-NS homologs from engaging in higher-order interactions with H-NS while maintaining the ability to form homomers. Members of the StpA clade are some of the most divergent in the oligomerization domain (Figure 3). The high degree of divergence may explain a distinguishing feature of StpA oligomers: they can interact with multiple DNA strands to form nucleoprotein structures whereas H-NS interacts with a maximum of two DNA strands to form bridges (33). Moreover, StpA or StpA:H-NS filaments were shown to have a stronger repressive effect than H-NS filaments (32). These results demonstrate that despite the degree of divergence of StpA and H-NS at key amino acid positions (Figure 3), they maintain an ability to form heteromers.

Previous experiments have examined how H-NS forms heteromers with Hha, a small H-NS N-terminal domain-like protein that binds H-NS, and how Hha impacts the DNA binding properties of H-NS (15,32). Hha blocks contacts between H-NS’s dimerization and DNA binding domains, which stimulates H-NS mediated DNA bridging and enhances repression of H-NS/Hha co-regulated genes (15,79,83,90). Arg12 is essential for H-NS binding with Hha (90,91); conservation of this amino acid in StpA, Hfp, Php, HlpC and HppX may allow Hha to interact with and influence the activity of H-NS homologs.

The amino acids Q112, G113 and R114 form a ‘A/T-hook-like’ motif that interacts with the minor groove of double stranded DNA (34). These three positions are 100% conserved in all homolog clades except for HppX, which has highly divergent sequences at these positions and across the flanking DNA binding region. Position 117 is divergent from H-NS in 94% of HppP sequences, and 100% divergent from the H-NS sequence in all other homolog clades (Figure 3D). The DNA binding domain is otherwise highly conserved across all homologs except HppX, suggesting that the newly discovered H-NS homologs share an ability to bind the same DNA targets. This could reinforce heteromer binding to DNA, or shared DNA targets could result in competition between H-NS and homologs in the same cell. The high degree of divergence between H-NS and the homologs at the DNA binding linker region may confer unique DNA binding and regulatory properties to the different members of the H-NS family of proteins. It has been previously observed that the rigid linker region of Sfh (HppH) explains differences in DNA binding specificity compared to H-NS (92).

### Implications of H-NS homolog diversity for understanding transcriptional regulation

The existence of an unexpectedly wide diversity of H-NS homologs and the varied distribution of these proteins among closely related bacterial strains presents intriguing questions about how cytoplasmic pools of H-NS proteins are coordinated, and how variable composition of the H-NS pool can in turn coordinate gene expression. The results from proteome and transcription quantification confirmed that H-NS is a dominant repressor of its homologs. Considering that only H-NS is core to all Enterobacterales genomes, it is expected to be deeply integrated and less susceptible to perturbation by its homologs.

H-NS deletion mutants are unlikely to persist in nature, yet their creation in the lab has been widely used to characterize H-NS functions. It is important to note that *hns* mutants are highly prone to compensatory mutations (2,70). For example, uncontrolled SPI-1 expression in an Δ*hns* mutant is deleterious; thus, targeted deletion or natural loss of SPI-1 or *hilD* can restore fitness in a Δ*hns* mutant (70). In Δ*hns*, StpA appears to evolve to become more H-NS-like, presumably to assume H-NS DNA binding and bridging activities. It is striking in our results that only Sfh is naturally equipped to regulate *hilD* expression if H-NS is removed from the system. These findings suggest that there will be no simple rules to predict the effects of the horizontal acquisition of an H-NS homolog. Rather, the effects will depend on both the genetic constituents and the growth phase (93).

The unexpected diversity of plasmid-borne H-NS homologs presents a wealth of research avenues to explore fundamental biology of plasmids, dynamics of gene transfer, and integration of horizontally acquired regulators into resident gene networks. As a central regulator of cell physiology, the study of H-NS underscores the importance of cellular physiology and environmental conditions when interpreting experiments that address network interactions and integration.

## DATA AVAILABILITY

All raw data from mass spectrometry is deposited at dryad.org, and is accessible through: https://doi.org/10.5061/dryad.t1g1jwt0j.

## Supplementary Material

gkaa709_Supplemental_Files
