# Supplementary material for: Redefining the H-NS protein family: a diversity of specialized core and accessory forms exhibit hierarchical transcriptional network integration
Source: Nucleic Acids Res. 2020 Sep 7;48(18):10184–98. doi: 10.1093/nar/gkaa709 (PMC7544231; doi:10.1093/nar/gkaa709)
Supplement: gkaa709_Supplemental_Files [file gkaa709_supplemental_files.zip › Supplementary Figures.pdf]

SUPPLEMENTARY TABLES - Available in separate Excel xlsx file.

Table S1. Chromosomal H-NS homolog accession numbers

Table S2. NCBI Microbial Plasmid database H-NS homologs

Table S3. PLSDB plasmids containing H-NS homologs

Table S4. Categorization of H-NS homologs in PLSDB according to the protein clades defined in this study.

Table S5. Plasmids with two H-NS homologs

Table S6. Oligonucleotide primers used in this study

Table S7. Amino acid positions in H-NS for which functions have been experimentally determined

SUPPLEMENTARY FIGURES LEGENDS

Figure S1. Bioinformatic workflow to identify H-NS homologs in Enterobacteriaceae genomes.

Figure S2. Rooted phylogenetic classification of H-NS family proteins in Enterobacteriaceae. Chromosomally-encoded (A) and plasmid-encoded clades (B) are indicated with the following colours: H-NS (green), StpA (red), Hfp (blue), HlpC (teal), HlpP (purple), HppP (magenta), HppX (orange), HppH (pink), HppE (grey), HppR (grey) and HppF (gold). Names of species and plasmids encoding H-NS family proteins are shown at branch tips. Phylogenies are derived from maximum likelihood estimates of evolutionary relationships, bootstrap scores > 70% are shown and the scale bar indicates the number of substitutions per site. Bren = Brenneria, C. fr = Citrobacter freundii, C. rod = Citrobacter rodentium, D. dad = Dickeya dadantii, E.c. = Escherichia coli, E. ferg = Escherichia fergusonii, Ent = Enterobacter, E.asb = Enterobacter asburiae E. dis = Enterobacter dissolvans, E. clo = Enterobacter cloaceae, E. hor = Enterobacter hormaechei, E. rad = Enterobacter radicincitans, E. Rog = Enterobacter roggenkampii Er. amy = Erwinia amylovora, Er. Per = Erwinia persicina, Er. Pir = Erwinia piriflorinigrans, Er. pyr = Erwinia pyrifoliae, Er. tra = Erwinia tracheiphila, K. ox = Klebsiella oxytoca, K. pn = Klebsiella pneumoniae, K. var = Klebsiella variicola Pant = Pantoea, P. agg = Pantoea agglomerans, P. ana = Pantoea ananatis, P. dis = Pantoea dispersa, P. stew = Pantoea stewartii, P. vag = Pantoea vagans, P. was = Pectobacterium wasabiae, P. atr = Pectobacterium atrosepticum, R. aq = Rahnella aquatilus, S.e. = Salmonella enterica, S. Cub = Salmonella enterica sv Cubana, S. Dub = Salmonella enterica sv Dublin, S. Ty = Salmonella enterica sv Typhi, S. Tym = Salmonella enterica sv Typhimurium, S. Ent = Salmonella enterica sv Enteritidis, S. Hei = Salmonella enterica sv Heidelberg, S. Hvi = Salmonella enterica sv Hvittingfoss, Sh. fle = Shigella flexneri, S. glo = Sodalis glossinidius, V. ch = Vibrio cholerae, Y. pes = Yersinia pestis, Y. pseu = Yersinia pseudotuberculosis.

Figure S3. Phylogenetic comparisons of StpA, H-NS and Hfp. A) An H-NS phylogeny resolves monophyletic clades of Enterobacteriaceae and Pectobacteriaceae H-NS proteins, congruent with whole genome phylogenies. The polyphyly of Erwiniaceae and Yersiniaceae H-NS arises from nodes with <45% bootstrap support, indicating that the low resolving power of H-NS phylogeny remains congruent with the bacterial phylogenies. Bootstrap values above 50% are indicated. B) In Enterobacteriaceae, the StpA and H-NS cladograms are fully congruent with the whole genome phylogeny. The bolded values below branches in the H-NS tree indicate bootstrap values from the whole genome phylogeny available at PATRIC (www.patricbrc.org). In Enterobacteriaceae and Pectobacteriaceae, the incongruence between the Hfp and H-NS phylogenies are explained as cases of horizontal transfer of the *hfp* gene. Strains that appear to have horizontally acquired *hfp* are indicated with grey fill. Strains of *E. coli* that lack Hfp are not shown. Bootstrap values above 50% are indicated, whereas branches below 50 % support are collapsed to polytomies.

Figure S4. Sequence logo analysis of amino acid conservation among H-NS homologs. A) A sequence logo showing the conserved residues in the 178 unique sequences upon which the phylogenies in Figure 1B and Figure S2 are based. Residues are coloured according to charge: positive (blue) and negative (red). Amino acid position numbering on the x-axis is according to the multiple sequence alignments. *Salmonella* H-NS position numbers are provided as per position numbering in Figure 3; triangles indicate the experimentally studied positions in Figure 3. B) Alignment of sequence logos for each protein clade in Figure 3. Residues are coloured according to charge: positive (blue) and negative (red). *Salmonella* H-NS position numbers are provided as per position numbering in Figure 3.

Figure S5. Regulation of *Salmonella* virulence gene expression by H-NS family proteins. Gene expression was quantified for SPI-1 (A), SPI-2 (B), and SPI-5 (C) genes in wildtype strain EN1660 and Δ*hns,* Δ*stpA,* Δ*hfp* mutants during exponential phase growth. Unlike Figure 5, here all gene expression data is normalized either to wildtype in the absence of pSf-R27 (right) or to wildtype cells containing pSf-R27. Because of experimental design, expression in the presence of pSf-R27 could not be directly compared to gene expression in cells without pSf-R27.

Workflow 1

	I	II
**Goal**	**Find plasmid-borne H-NS homologs**	**Find Hfp homologs in any Enterobacterales genome**
Query sequences	H-NS (*E. coli*)<br>StpA (*E. coli*)<br>Sfh (*S. flexneri*)	Hfp (*E. coli*)
BLAST Type	tblastn	blastp
Database and subjects	NCBI Microbial Plasmid Database	NCBI Microbial Protein Database
Data filtering	>50% similarity over full-length	>90% similarity over full-length

Workflow 2

	I	II
**Goal**	**Find chromosomal H-NS homologs in genomes with a plasmid-borne H-NS homolog**	**Find H-NS homologs in genomes with Hfp**
Query sequences	H-NS (*E. coli*)<br>StpA (*E. coli*)<br>Sfh (*S. flexneri*)	H-NS (E. coli)<br>StpA (E. coli)
BLAST Type	blastp	blastp
Database and subjects	Genomes represented in Microbial Plasmid Database	Enterobacterales genomes with Hfp
Data filtering	>50% similarity over full-length	>50% similarity over full-length

Phylogenetic Analysis

Remove identical sequences; align proteins

Figure 1, Figure S2, Figure S3

Supplementary Figure S1

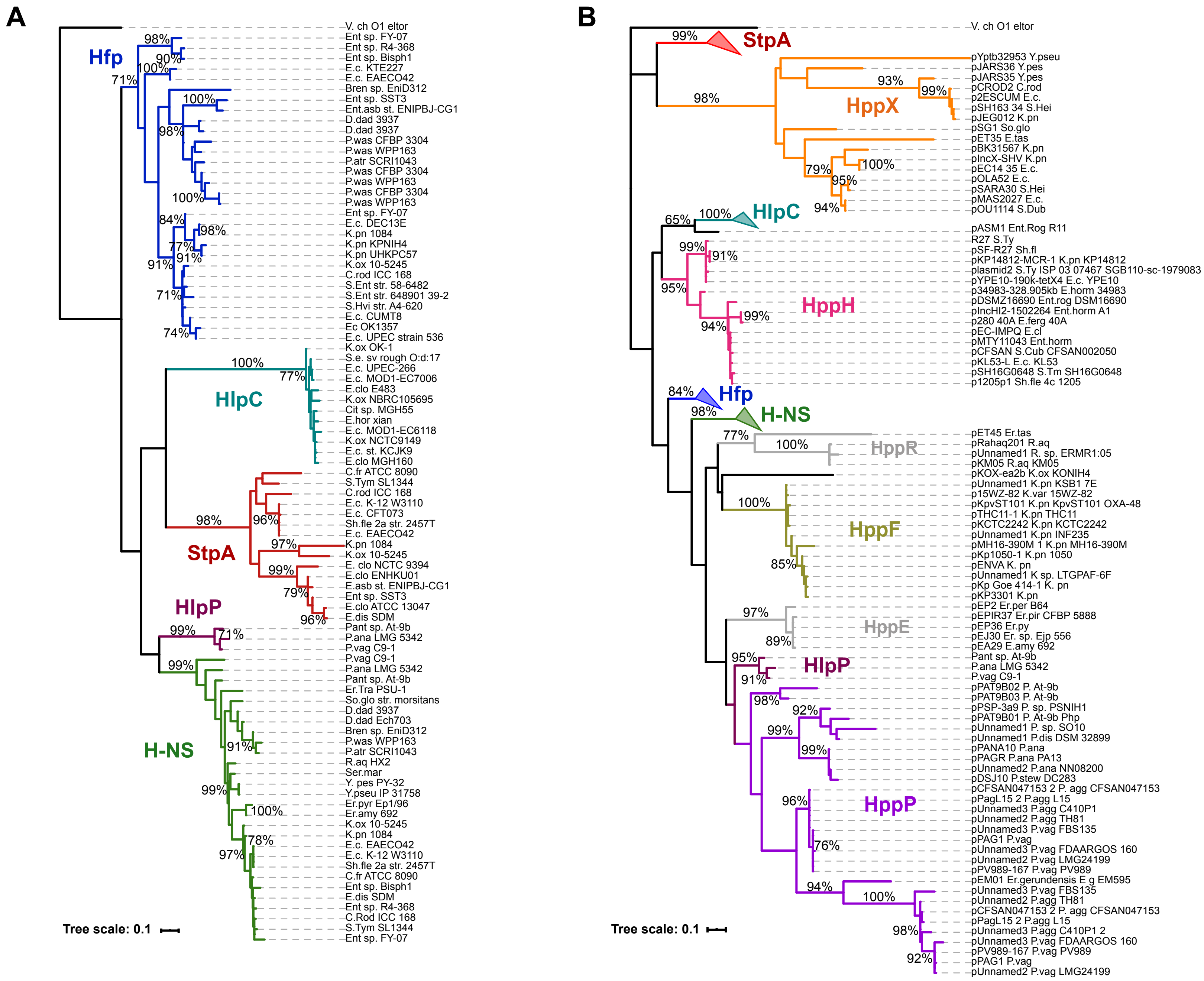

Supplementary Figure S2

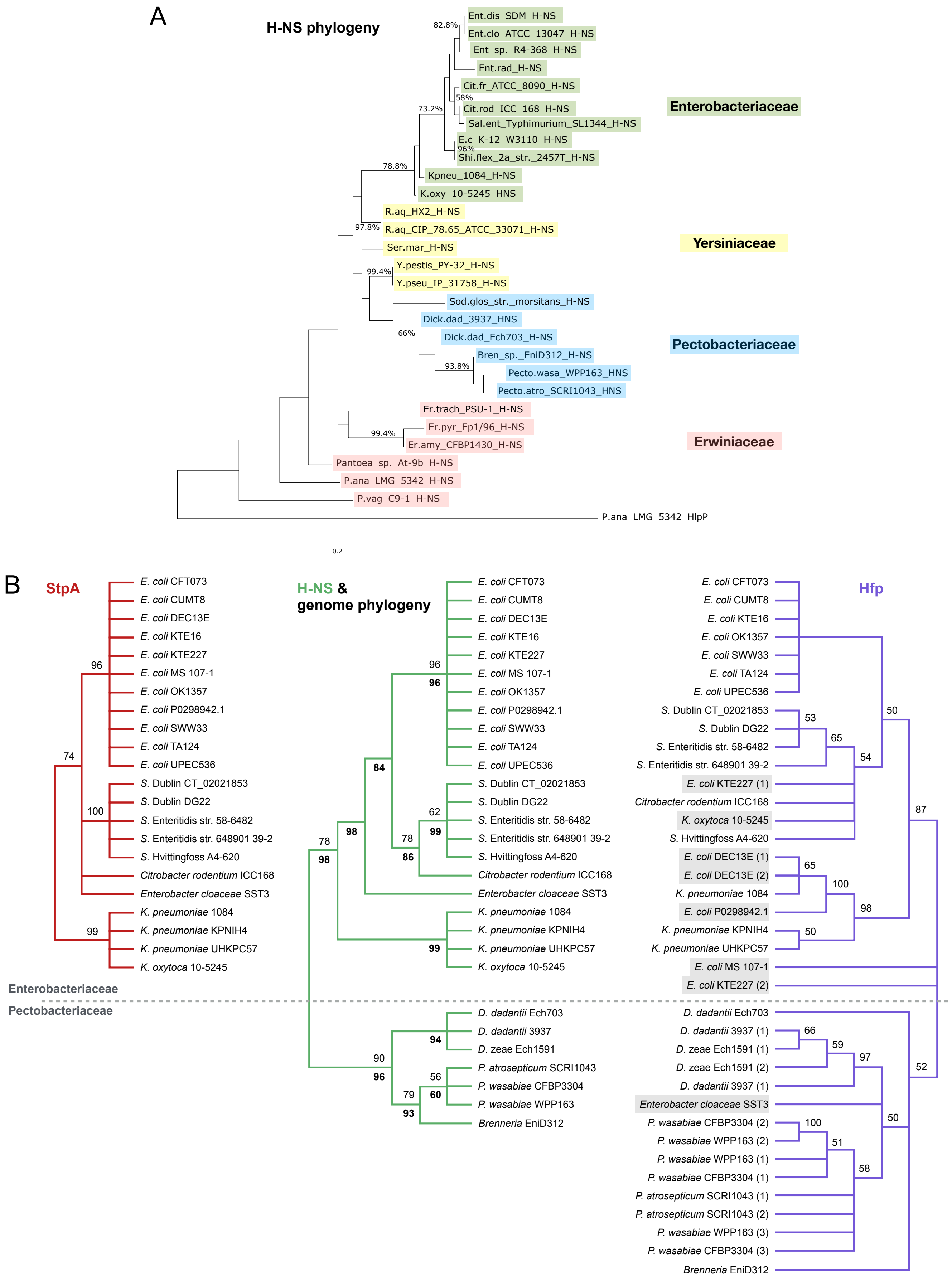

Supplementary Figure S3

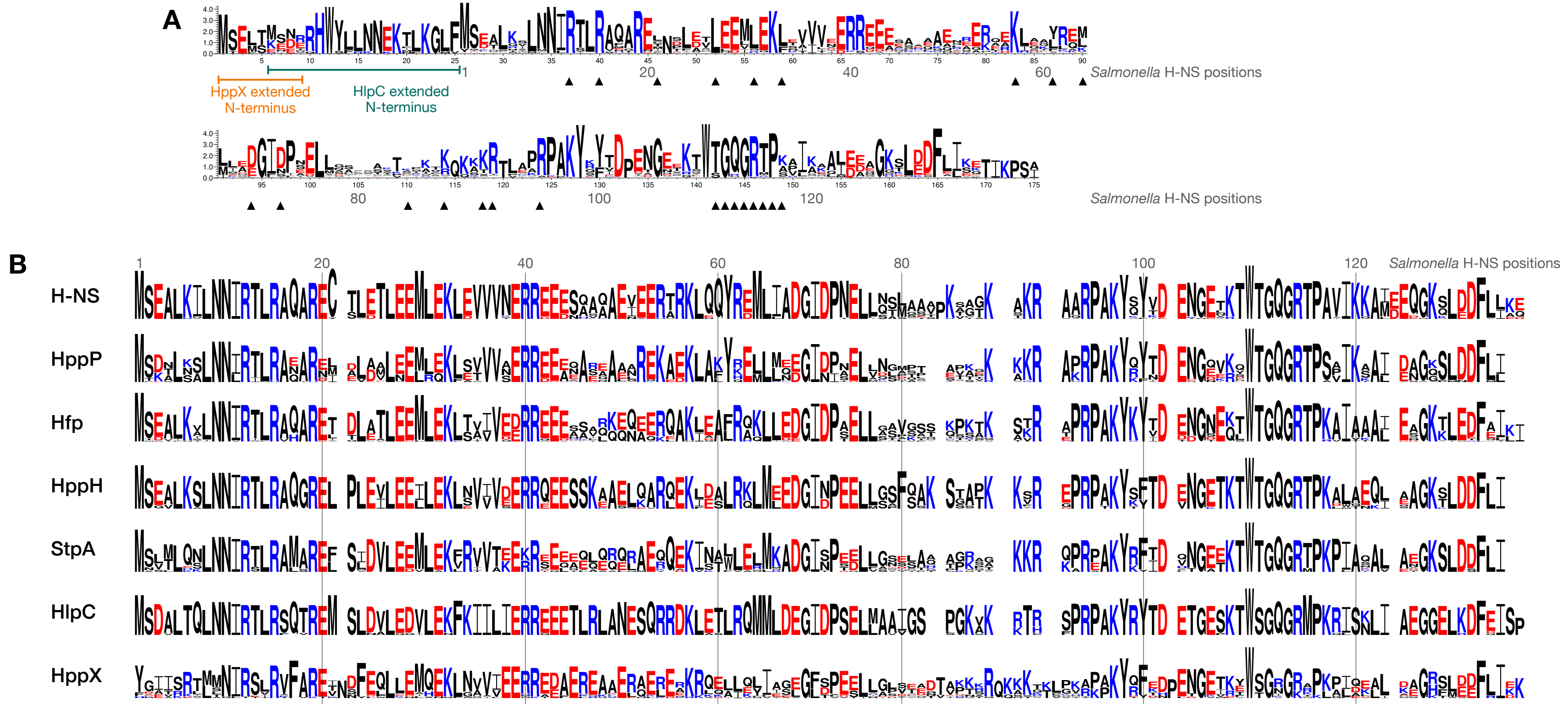

Supplementary Figure S4

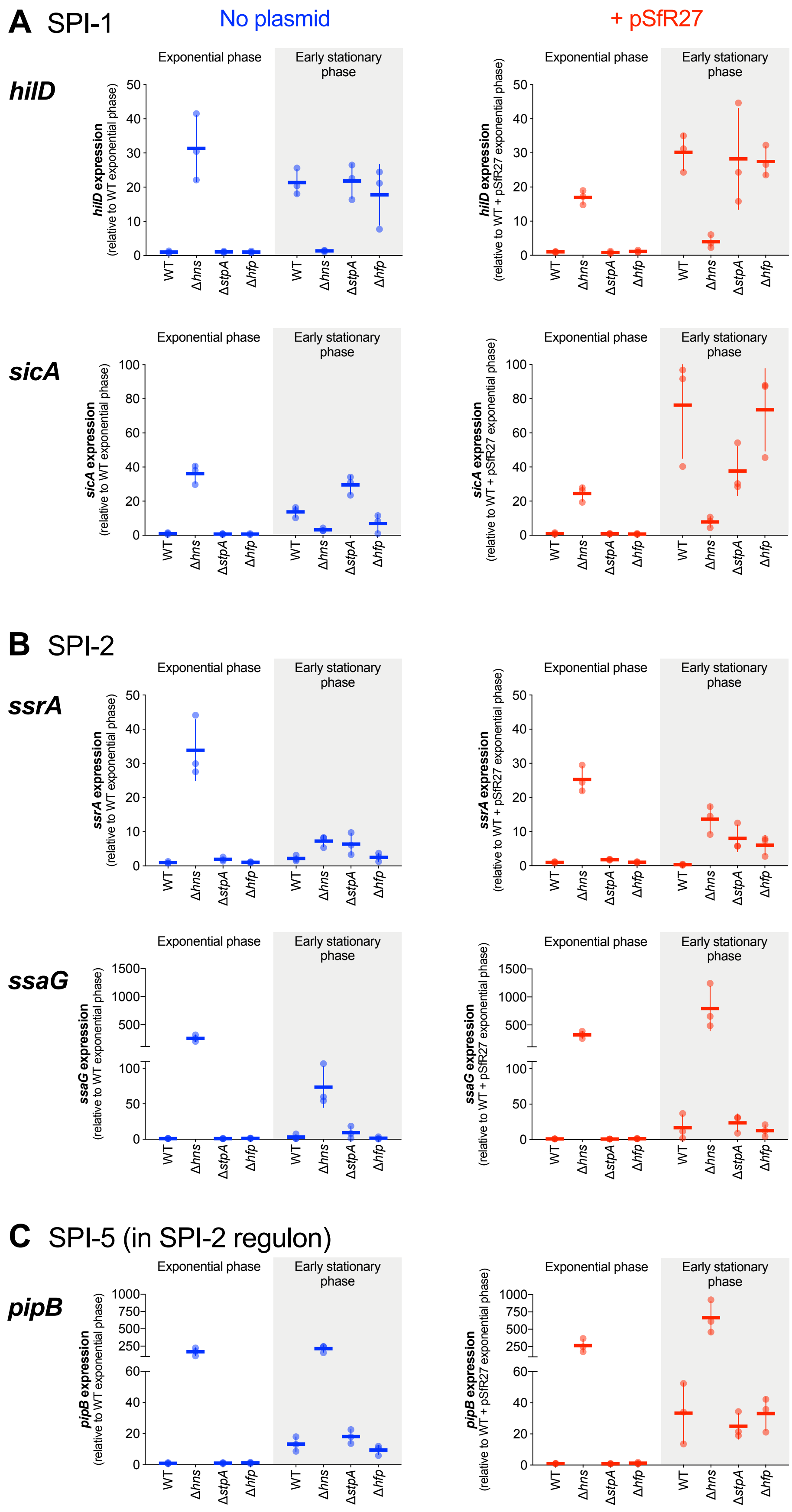

Supplementary Figure S5
